# Supplementary material for: A Likelihood Approach for Real-Time Calibration of Stochastic Compartmental Epidemic Models
Source: PLoS Comput Biol. 2017 Jan 17;13(1):e1005257. doi: 10.1371/journal.pcbi.1005257 (PMC5240920; doi:10.1371/journal.pcbi.1005257)
Supplement: S1 File — (TAR.GZ) [file pcbi.1005257.s014.tar.gz › HSPH_Online-SI-Revision/PF08/n100-severe/n100-severe_PF_table-4.pdf]

1	R0	{1.65278, 9.06793}	{2.19036, 20.}	{{2.12272, 20.}, {2.24591, 20.}}	{32.5261, 120.558}	inf	4882.	9675.33	{8980.87, 10 242.4}	98.1837	0.296537	newly	4385.	3842.48	{3546., 4098.}	12.3722	0.0579653	Reff	1.41083	1.75217	{1.72949, 1.77532}	24.1945	ar	66 342.	92 976.5	{91 700., 94 242.}	40.1473
2	R0	{1.4321, 3.80644}	{2.99538, 19.9738}	{{3., 20.}, {3., 20.}}	{109.16, 424.737}	inf	1776.	8201.33	{7626.3, 8927.42}	361.787	0.66359	newly	4438.	3831.35	{3547., 4167.}	13.6694	0.0645366	Reff	1.34796	2.65894	{2.62799, 2.68783}	97.2561	ar	54 217.	97 645.5	{97 343., 97 931.}	80.1013
3	R0	{1.68388, 4.76028}	{2.99902, 19.6896}	{{3., 19.4414}, {3., 20.}}	{78.1015, 313.623}	inf	2116.	6934.13	{6589.45, 7271.65}	227.7	0.514802	newly	4947.	3335.55	{3150., 3578.}	32.5743	0.171765	Reff	1.59182	2.69664	{2.70008, 2.71583}	69.4061	ar	68 299.	98 276.9	{97 587., 98 976.}	43.8921
4	R0	{1.66014, 6.95383}	{2.91166, 20.}	{{2.82533, 20.}, {3., 20.}}	{75.3865, 187.611}	inf	3294.	9587.64	{8760.36, 10 133.6}	191.064	0.463315	newly	4326.	4342.66	{3969., 4677.}	4.81923	0.0208418	Reff	1.51479	2.50494	{2.4574, 2.57328}	65.3653	ar	66 820.	97 274.4	{96 590., 97 722.}	45.5768
5	R0	{1.69984, 11.2038}	{2.23997, 20.}	{{2.17591, 20.}, {2.30418, 20.}}	{31.7752, 78.5109}	inf	6557.	11 682.7	{10 730.7, 12 494.}	78.1711	0.250166	newly	4410.	4590.48	{4188., 4969.}	6.29977	0.0264631	Reff	1.38564	1.70238	{1.65953, 1.7514}	22.8589	ar	68 462.	94 289.6	{93 059., 95 453.}	37.7254
6	R0	{1.65876, 6.66217}	{2.73586, 20.}	{{2.67574, 20.}, {2.78853, 20.}}	{64.9342, 200.202}	inf	2507.	6807.81	{6350.41, 7341.55}	171.552	0.43316	newly	3575.	3063.65	{2851., 3293.}	14.3035	0.0678016	Reff	1.55026	2.45009	{2.40829, 2.49325}	58.0441	ar	67 105.	94 774.3	{94 123., 95 674.}	41.2328
7	R0	{1.69966, 5.89996}	{2.94194, 19.7909}	{{2.83129, 20.}, {3., 20.}}	{73.09, 235.44}	inf	2040.	6530.11	{5402.08, 7118.54}	220.103	0.503034	newly	3565.	3112.57	{2726., 3397.}	12.6909	0.0609108	Reff	1.61145	2.68227	{2.6271, 2.7595}	66.4511	ar	69 396.	97 321.5	{96 846., 97 994.}	40.2408
8	R0	{1.53428, 6.3734}	{2.15666, 17.6615}	{{2.11478, 16.8011}, {2.18969, 18.3945}}	{40.5652, 177.112}	inf	2925.	7104.78	{6733.06, 7513.3}	142.898	0.384902	newly	3946.	3357.75	{3136., 3603.}	14.9075	0.0707093	Reff	1.38868	1.90332	{1.87046, 1.92955}	37.0601	ar	59 855.	89 518.7	{88 329., 90 333.}	49.5593
9	R0	{1.58939, 3.48608}	{2.44721, 18.7794}	{{1.64126, 15.3309}, {2.65397, 20.}}	{54.3987, 438.697}	inf	458.	1819.51	{866.702, 2211.33}	297.273	0.581434	newly	1645.	873.92	{695., 1021.}	46.8742	0.281023	Reff	1.56577	2.37238	{1.60492, 2.57344}	52.0025	ar	62 575.	88 970.3	{69 042., 93 735.}	42.3919
10	R0	{1.58528, 5.5593}	{1.9698, 20.}	{{1.96219, 20.}, {1.97583, 20.}}	{24.2557, 259.758}	inf	2476.	5010.	{4844.61, 5059.99}	102.343	0.306015	newly	4136.	2006.79	{1943., 2068.}	51.4799	0.31424	Reff	1.4648	1.78667	{1.77778, 1.7918}	21.9737	ar	63 039.	84 208.7	{83 751., 84 599.}	33.5819
11	R0	{1.58305, 4.62721}	{3., 20.}	{{3., 20.}, {3., 20.}}	{89.5076, 332.226}	inf	1827.	6700.94	{6371., 7146.97}	266.773	0.564017	newly	3948.	3173.07	{2996., 3392.}	19.6284	0.0953514	Reff	1.50095	2.72778	{2.70673, 2.74155}	81.7363	ar	63 222.	96 978.4	{96 722., 97 257.}	53.3934
12	R0	{1.70234, 9.34626}	{1.99708, 20.}	{{1.98554, 20.}, {2.00496, 20.}}	{17.314, 113.989}	inf	5054.	6806.79	{6661.76, 6912.58}	34.6812	0.129263	newly	4443.	2700.01	{2618., 2778.}	39.23	0.216428	Reff	1.46651	1.7389	{1.73027, 1.74499}	18.5739	ar	68 213.	85 707.8	{85 227., 86 143.}	25.6472
13	R0	{1.68556, 9.74954}	{2.21863, 20.}	{{2.13187, 20.}, {2.2849, 20.}}	{31.6255, 105.138}	inf	5632.	10 432.6	{9832.77, 11 030.8}	85.2386	0.267292	newly	4572.	4114.63	{3903., 4342.}	10.0405	0.0463859	Reff	1.41078	1.72476	{1.68776, 1.75319}	22.2557	ar	68 426.	92 996.5	{90 911., 94 478.}	35.9081
14	R0	{1.59876, 9.78897}	{2.17023, 19.8754}	{{2.05652, 20.}, {2.24832, 20.}}	{35.7445, 103.039}	inf	4188.	8462.32	{7640.08, 9023.4}	102.061	0.304003	newly	3466.	3406.36	{3104., 3600.}	4.7236	0.0212258	Reff	1.39393	1.77701	{1.74213, 1.82552}	27.4825	ar	63 553.	93 054.2	{91 250., 94 746.}	46.4198
15	R0	{1.43577, 5.12819}	{2.18269, 20.}	{{2.14422, 20.}, {2.22751, 20.}}	{52.022, 290.001}	inf	1915.	6598.67	{6265.07, 6940.39}	244.578	0.536832	newly	3233.	2721.37	{2548., 2903.}	15.8252	0.075471	Reff	1.33374	1.94557	{1.89506, 1.97314}	45.8726	ar	53 259.	91 131.8	{90 218., 91 959.}	71.1105
16	R0	{1.58866, 8.73191}	{2.15999, 20.}	{{2.12296, 20.}, {2.18983, 20.}}	{35.9629, 129.045}	inf	3460.	6747.96	{6236.53, 7334.69}	95.0277	0.289331	newly	3246.	2724.4	{2495., 2991.}	16.069	0.0770067	Reff	1.43615	1.82941	{1.80637, 1.87094}	27.3833	ar	64 349.	90 210.7	{89 516., 90 903.}	40.1898
17	R0	{1.43012, 3.8163}	{2.79032, 19.4955}	{{2.51209, 20.}, {2.94197, 20.}}	{95.1108, 410.848}	inf	970.	4657.39	{4140.46, 5137.68}	380.143	0.675716	newly	2605.	2231.6	{1955., 2438.}	14.7209	0.0709671	Reff	1.37832	2.61471	{2.3754, 2.74849}	89.7026	ar	53 353.	96 060.	{93 897., 98 146.}	80.0461
18	R0	{1.47965, 5.16472}	{2.36453, 16.7738}	{{1.91462, 11.8122}, {2.72069, 20.}}	{59.8032, 224.776}	inf	2352.	7218.96	{4854.34, 9163.34}	206.928	0.471465	newly	4058.	3763.43	{3402., 4118.}	9.02785	0.041564	Reff	1.36212	2.10122	{1.75393, 2.36834}	54.2611	ar	56 086.	91 098.4	{82 808., 97 219.}	62.4263
19	R0	{1.57191, 5.2314}	{2.84468, 20.}	{{2.75711, 20.}, {2.90075, 20.}}	{80.9696, 282.307}	inf	3247.	11 356.9	{10 877.9, 11 758.8}	249.765	0.54356	newly	5657.	4982.38	{4746., 5221.}	11.9254	0.0554352	Reff	1.41239	2.34992	{2.29189, 2.39968}	66.3784	ar	63 119.	96 929.9	{95 995., 98 001.}	53.567
20	R0	{1.54435, 5.32911}	{2.28998, 19.926}	{{2.27137, 20.}, {2.31018, 20.}}	{48.281, 273.909}	inf	2562.	6962.75	{6837.39, 7129.04}	171.77	0.433788	newly	4511.	2938.03	{2828., 3036.}	34.8697	0.186516	Reff	1.42398	2.05329	{2.04487, 2.06698}	44.1937	ar	61 870.	93 676.3	{93 316., 94 112.}	51.4083
21	R0	{1.57932, 4.90031}	{2.95153, 19.9718}	{{2.84846, 20.}, {3., 20.}}	{86.8863, 307.561}	inf	1638.	6127.79	{5474.6, 7134.15}	274.102	0.570949	newly	3321.	2902.08	{2597., 3369.}	13.3532	0.0638146	Reff	1.50468	2.70554	{2.63122, 2.76314}	79.8084	ar	63 595.	97 227.5	{96 664., 97 825.}	52.8854
22	R0	{1.47044, 4.07694}	{2.08637, 16.7775}	{{2.03604, 16.2752}, {2.13151, 17.3074}}	{41.8877, 311.522}	inf	941.	3172.27	{2859.58, 3426.54}	237.117	0.526364	newly	2369.	1627.88	{1465., 1749.}	31.2841	0.164153	Reff	1.4165	1.98172	{1.92798, 2.02434}	39.9019	ar	55 895.	85 792.	{84 490., 87 018.}	53.4878
23	R0	{1.64997, 3.34546}	{2.85906, 18.4581}	{{2.22386, 15.1186}, {3., 20.}}	{73.2795, 451.735}	inf	758.	3084.5	{2090.05, 3531.03}	306.926	0.602512	newly	3196.	1614.58	{1288., 1818.}	49.4812	0.299974	Reff	1.61331	2.67371	{2.14745, 2.8508}	65.7285	ar	66 404.	94 724.3	{87 790., 97 058.}	42.6485
24	R0	{1.67405, 4.50254}	{2.9132, 16.1881}	{{2.62942, 14.5262}, {3., 20.}}	{74.3317, 259.532}	inf	1073.	2773.79	{2381.79, 3252.42}	159.324	0.409201	newly	2825.	1857.9	{1305., 2374.}	34.2336	0.19362	Reff	1.63156	2.81141	{2.53966, 2.91261}	72.5826	ar	68 017.	95 656.8	{93 586., 97 189.}	40.8307
25	R0	{1.50663, 3.1286}	{2.96127, 16.7295}	{{2.96627, 14.6245}, {3., 20.}}	{96.5492, 434.729}	inf	639.	2806.59	{2436.87, 3293.17}	339.216	0.639331	newly	2373.	1820.6	{1336., 2444.}	24.9414	0.133441	Reff	1.47669	2.8553	{2.70419, 2.91783}	93.3576	ar	58 823.	96 562.9	{96 165., 97 455.}	64.1584
26	R0	{1.69822, 6.34992}	{2.1718, 17.6652}	{{2.16888, 17.6023}, {2.17478, 17.7397}}	{27.8866, 178.196}	inf	2762.	5146.46	{5134.9, 5179.27}	86.3308	0.270222	newly	4173.	2483.99	{2427., 2538.}	40.4747	0.225387	Reff	1.57542	1.99599	{1.99361, 1.99846}	26.6959	ar	69 816.	88 886.8	{88 642., 89 153.}	27.3158
27	R0	{1.55869, 4.92988}	{2.73226, 20.}	{{2.71716, 20.}, {2.74411, 20.}}	{75.2921, 305.689}	inf	1917.	7557.48	{7494.51, 7597.59}	294.235	0.595748	newly	3834.	3400.33	{3340., 3480.}	11.3112	0.0521874	Reff	1.46882	2.44193	{2.429, 2.45152}	66.2514	ar	62 344.	96 753.8	{96 534., 96 978.}	55.1934
28	R0	{1.46085, 6.06899}	{2.19264, 16.0559}	{{1.82847, 11.1619}, {2.53181, 20.}}	{50.0931, 164.557}	inf	3200.	8890.38	{6191.36, 11 644.3}	177.824	0.425976	newly	4410.	4603.86	{4233., 4930.}	6.27075	0.0262519	Reff	1.29135	1.8363	{1.58269, 2.06682}	42.2006	ar	55 484.	86 977.	{78 505., 94 081.}	56.7604
29	R0	{1.63017, 6.25164}	{2.92891, 20.}	{{2.87138, 20.}, {2.97195, 20.}}	{79.669, 219.916}	inf	3520.	10 524.7	{10 066.1, 10 940.9}	198.998	0.475373	newly	5090.	4737.42	{4497., 4969.}	7.13517	0.0324124	Reff	1.47729	2.48441	{2.43279, 2.52694}	68.1733	ar	66 231.	98 564.3	{97 599., 99 484.}	48.8189
30	R0	{1.45173, 5.68165}	{2.17728, 20.}	{{2.16576, 20.}, {2.18806, 20.}}	{49.9784, 252.01}	inf	2741.	6691.41	{6572.82, 6789.05}	144.123	0.387507	newly	3975.	2690.11	{2580., 2766.}	32.3243	0.16972	Reff	1.31862	1.81107	{1.80168, 1.81971}	37.3457	ar	55 280.	89 946.1	{89 412., 90 348.}	62.7101
31	R0	{1.55808, 2.77316}	{2.85398, 16.4222}	{{2.35731, 14.6498}, {3., 20.}}	{83.2642, 492.183}	inf	583.	2575.26	{2156.32, 2847.93}	341.725	0.639485	newly	3043.	1676.06	{1139., 2159.}	44.9208	0.271465	Reff	1.52792	2.76185	{2.28436, 2.92385}	80.8391	ar	60 940.	95 299.3	{90 214., 97 344.}	56.3883
32	R0	{1.56797, 3.90327}	{2.89575, 19.6239}	{{2.38683, 20.}, {3., 20.}}	{84.6812, 402.754}	inf	984.	3915.19	{3142.92, 4552.34}	297.885	0.595261	newly	2775.	1922.26	{1511., 2210.}	30.7294	0.163261	Reff	1.52355	2.74512	{2.28996, 2.85677}	80.1793	ar	62 228.	95 866.7	{91 440., 97 214.}	54.0572
33	R0	{1.70027, 5.43261}	{3., 19.8492}	{{3., 20.}, {3., 20.}}	{76.4426, 265.372}	inf	2721.	7511.46	{7214.92, 7692.12}	176.055	0.440683	newly	5236.	3579.4	{3418., 3664.}	31.6387	0.165801	Reff	1.58887	2.70035	{2.69244, 2.73892}	69.9544	ar	68 798.	100 446.	{99 596., 101 070.}	46.0017
34	R0	{1.61668, 6.86192}	{1.93809, 20.}	{{1.93081, 20.}, {1.94297, 20.}}	{19.8806, 191.464}	inf	3615.	5086.76	{5069.3, 5111.57}	40.7127	0.148325	newly	4645.	2022.61	{1965., 2071.}	56.4562	0.361166	Reff	1.44922	1.74779	{1.73946, 1.75356}	20.6017	ar	64 693.	83 158.2	{82 741., 83 509.}	28.5427
35	R0	{1.55955, 3.45071}	{2.69808, 18.1269}	{{2.16553, 14.7628}, {3., 20.}}	{73.0503, 425.308}	inf	643.	2501.44	{1718.54, 3142.22}	289.027	0.575629	newly	2408.	1305.1	{1051., 1562.}	45.8015	0.27342	Reff	1.53037	2.57303	{2.08104, 2.86698}	68.2116	ar	61 783.	92 601.7	{86 200., 96 956.}	49.8822
36	R0	{1.65068, 4.91307}	{2.77688, 19.2833}	{{2.53171, 19.8062}, {2.94526, 20.}}	{68.2264, 292.49}	inf	1136.	3733.13	{3304.48, 4230.84}	228.621	0.507682	newly	2506.	1822.33	{1575., 2013.}	27.2813	0.140681	Reff	1.60096	2.64304	{2.42584, 2.79574}	65.0906	ar	66 453.	95 999.8	{94 598., 98 629.}	44.4627
37	R0	{1.46131, 3.06714}	{2.98939, 15.4094}	{{3., 15.1583}, {3., 15.984}}	{104.569, 402.403}	inf	856.	4133.59	{3751.91, 4278.69}	382.896	0.682937	newly	3280.	2879.58	{2308., 3118.}	12.3335	0.0605513	Reff	1.41952	2.79323	{2.78568, 2.83015}	96.773	ar	56 452.	95 816.3	{95 552., 96 082.}	69.7306
38	R0	{1.71034, 5.2546}	{3., 20.}	{{3., 20.}, {3., 20.}}	{75.4037, 280.619}	inf	2107.	6668.27	{6362.36, 6866.91}	216.482	0.500049	newly	4350.	3151.52	{2992., 3265.}	27.5513	0.140301	Reff	1.62197	2.71437	{2.70539, 2.72823}	67.3505	ar	69 816.	96 600.6	{96 361., 96 863.}	38.3646
39	R0	{1.43565, 5.57989}	{2.08197, 14.9132}	{{1.88742, 12.8534}, {2.493, 20.}}	{45.0192, 167.267}	inf	2976.	7811.99	{6327.46, 11 032.8}	162.5	0.409112	newly	4327.	4385.27	{3990., 4691.}	5.03143	0.0217133	Reff	1.28285	1.81331	{1.67647, 2.09199}	41.3496	ar	54 407.	88 625.7	{84 196., 96 842.}	62.8939
40	R0	{1.40327, 4.00764}	{2.86345, 19.2195}	{{2.45592, 17.3019}, {3., 20.}}	{104.055, 379.572}	inf	1552.	7480.71	{6134.01, 8621.68}	382.005	0.677647	newly	3528.	3612.48	{3123., 4017.}	7.15079	0.0306908	Reff	1.32289	2.58336	{2.24214, 2.72103}	95.2814	ar	51 976.	97 628.5	{94 757., 99 295.}	87.8338
41	R0	{1.63612, 4.26741}	{3., 15.9182}	{{3., 15.4163}, {3., 16.2793}}	{83.3606, 273.017}	inf	1646.	5721.89	{5303.53, 6017.81}	247.624	0.540596	newly	4452.	3766.69	{3576., 4000.}	15.3933	0.0734274	Reff	1.56264	2.79884	{2.77898, 2.817}	79.1096	ar	65 029.	98 839.8	{98 156., 99 318.}	51.9935
42	R0	{1.57426, 6.50271}	{2.19506, 20.}	{{2.15143, 20.}, {2.22404, 20.}}	{39.4342, 207.564}	inf	3056.	6982.57	{6487.63, 7163.06}	128.487	0.358525	newly	4138.	2878.6	{2658., 2988.}	30.435	0.158038	Reff	1.43171	1.95369	{1.90517, 1.97303}	36.4587	ar	62 522.	93 011.6	{91 370., 93 744.}	48.7662
43	R0	{1.37818, 5.37636}	{2.18702, 20.}	{{2.13954, 20.}, {2.22926, 20.}}	{58.6888, 271.999}	inf	2468.	8106.99	{7387.52, 8654.43}	228.484	0.515582	newly	3812.	3281.13	{2950., 3535.}	13.9263	0.0661637	Reff	1.24355	1.85535	{1.81856, 1.88792}	49.1983	ar	50 098.	92 047.9	{91 281., 92 825.}	83.7357
44	R0	{1.66606, 4.92641}	{2.93754, 19.6769}	{{3., 20.}, {3., 20.}}	{76.3244, 299.416}	inf	1205.	3913.85	{3576.4, 4572.12}	224.8	0.506688	newly	2554.	1920.21	{1731., 2227.}	24.8156	0.126718	Reff	1.61485	2.78489	{2.81395, 2.85713}	72.4572	ar	67 066.	96 017.9	{96 093., 97 039.}	43.1693
45	R0	{1.623, 5.9449}	{2.7615, 19.9924}	{{2.6809, 20.}, {2.84335, 20.}}	{70.1481, 236.296}	inf	2171.	6488.87	{5996.86, 6961.97}	198.889	0.474859	newly	3513.	2944.09	{2752., 3160.}	16.1944	0.077375	Reff	1.52557	2.48502	{2.42312, 2.57072}	62.8912	ar	64 554.	95 457.1	{94 384., 96 621.}	47.8717
46	R0	{1.65235, 5.51234}	{2.0931, 16.7175}	{{2.07256, 16.1725}, {2.12722, 17.0498}}	{26.6744, 203.273}	inf	1774.	3425.29	{3187.58, 3610.77}	93.0831	0.28522	newly	3211.	1760.43	{1631., 1870.}	45.175	0.261515	Reff	1.57561	1.97691	{1.95082, 2.00445}	25.4689	ar	66 509.	86 088.9	{85 492., 86 709.}	29.4394
47	R0	{1.62138, 8.82208}	{2.20537, 19.6403}	{{2.11101, 17.7446}, {2.26473, 20.}}	{36.0178, 122.627}	inf	5092.	11 310.6	{10 483.3, 11 988.8}	122.125	0.345892	newly	4726.	4587.56	{4272., 4848.}	4.3978	0.0197052	Reff	1.36283	1.76641	{1.68922, 1.80691}	29.613	ar	65 598.	92 638.7	{90 947., 93 928.}	41.2219
48	R0	{1.64073, 6.69261}	{2.87178, 19.9348}	{{2.79517, 20.}, {2.96634, 20.}}	{75.0306, 197.862}	inf	3000.	8778.44	{8011.8, 9397.27}	192.615	0.465301	newly	4095.	3991.05	{3677., 4302.}	5.53724	0.0246722	Reff	1.5074	2.49865	{2.43353, 2.5582}	65.7584	ar	66 438.	97 285.5	{96 388., 98 530.}	46.4305
49	R0	{1.65556, 3.796}	{2.63388, 16.2699}	{{1.9257, 11.5503}, {3., 20.}}	{59.1667, 328.606}	inf	673.	1724.07	{1007.59, 2352.25}	156.176	0.388322	newly	2370.	1063.2	{703., 1677.}	55.1392	0.370112	Reff	1.6296	2.52845	{1.8698, 2.92119}	55.2727	ar	66 575.	91 456.2	{80 342., 96 789.}	37.4012
50	R0	{1.56828, 3.65713}	{3., 16.0944}	{{3., 15.5095}, {3., 16.4715}}	{91.2924, 340.083}	inf	1346.	5513.59	{5236.14, 5850.52}	309.628	0.612004	newly	4132.	3589.67	{3417., 3847.}	13.1251	0.0621623	Reff	1.50552	2.81585	{2.79539, 2.83344}	87.0352	ar	62 761.	99 278.7	{98 662., 99 805.}	58.1853
